# Widespread neuronal hemokinin-1 expression in motor regions and its protective role in age-related impairment

**DOI:** 10.1007/s11357-025-01972-4

**Published:** 2025-11-12

**Authors:** Dávid Vince Simon, Katalin Rozmer, Eszter Kepe, Norbert Tóth, Krisztina Pohóczky, Orsolya Salamonné Mihály, Bibiána Török, Éva Renner, Miklós Palkovits, Zsuzsanna Helyes, Éva Borbély

**Affiliations:** 1 Department of Pharmacology and Pharmacotherapy, Medical School, University of Pécs, Pécs, Hungary; 2 Centre for Neuroscience, University of Pécs, Pécs, Hungary; 3 Department of Pharmaceutical Chemistry, Faculty of Pharmacy, University of Pécs, Pécs, Hungary; 4 Department of Pharmacology, Faculty of Pharmacy, University of Pécs, Pécs, Hungary; 5 Department of Pathology, Saint Borbala Hospital, Tatabánya, Budapest, Hungary; 6 Human Brain Research Laboratory, HUN-REN Institute of Experimental Medicine, Budapest, Hungary; 7 Human Brain Tissue Bank, Semmelweis University, Budapest, Hungary; 8 Chronic Pain Research Group, Hungarian Research Network, University of Pécs, Pécs, Hungary; 9 National Laboratory for Drug Research and Development, Budapest, Hungary

**Keywords:** Tachykinins, Rotarod, Static bar, Horizontal bar, Grid test, Sex differences

## Abstract

Hemokinin-1 (HK-1) regulates several processes in the central and peripheral nervous systems, including musculoskeletal functions; however, its involvement in age-related motor coordination and muscle strength remains unknown. Therefore, these were investigated here with HK-1-deficient mice together with the expression patterns in the mouse and human motor coordination centers. Tac4 gene expression, encoding HK-1, was detected by RNAscope, and its age-dependent changes in the cerebellum and soleus muscle were analyzed by qPCR. The rotarod and static rod tests were used to measure motor coordination, while the horizontal bar and grid tests assessed muscle strength in 3–4 (young), 12 (middle-aged), and 18-month-old (old) C57BL/6 wildtype (WT) and HK-1-deficient male and female mice. HK-1 mRNA was abundant in motor cortices, basal ganglia, and cerebellum, showing a significant increase with age. Motor coordination gradually declined with aging in WT mice of both sexes. This age-dependent reduction in motor coordination was significantly enhanced by HK-1 deficiency in middle-aged males but not females. Additionally, muscle strength was also remarkably worse in old WT mice, with a significantly greater decline in males. HK-1 deletion resulted in improved muscle strength in middle-aged male mice, but interestingly worsened function in the old groups of both sexes. HK-1 exerts age- and sex-dependent effects on motor coordination with a predominantly protective action on aging-induced decline in males. This might be explained by its high expression in all motor regions of the brain. Meanwhile, its function in regulating muscle strength is more complex, with a protective role only evident in old age.

**Supplementary Information:**

The online version contains supplementary material available at https://doi.org/10.1007/s11357-025-01972-4.

## Introduction

Statistical analysis suggests that by 2050, 22% of the world’s population will be over 60 years old (https://www.who.int/news-room/fact-sheets/detail/ageing-and-health). Age-related changes in the human body make the daily life of the elderly society even more difficult. Such negative changes include, for example, changes in motor functions that can lead to physical limitations, accidents, and injuries. The decline in motor function consists, among others, of deterioration in motor coordination [1] and a decrease in muscle strength [2]. In old age, bimanual and multijoint movements become more difficult, such as walking or lifting and moving objects, which are essential for a whole life. Research has shown that the incidence of bone fractures and physical disabilities (i.e., impairment of body function or structure, activity, and participation limitations) increases with age. These, along with their associated potential complications, will place an additional burden on the health system [3, 4].

Motor behavior is regulated by a sophisticated neural network that originates in the cerebral cortex and terminates in the skeletal muscles in humans and mice. Various cortical centers, including the sensorimotor cortex, association cortex, basal ganglia, and cerebellum, interact intensively to coordinate the movements of different body parts to perform complex actions, such as running or climbing [5–7].

Hemokinin-1 (HK-1) belongs to the neuropeptide tachykinin family. Until 2000, this family had only three members, when HK-1 was first identified in B lymphocytes [8]. HK-1 is encoded by the tachykinin 4 (Tac4) gene in mice and is similar in many respects to the well-known member of the family, Substance P (SP). The chemical structures of the two neuropeptides are more than 50% identical; both of them act as agonists at the NK1 receptor. In addition to the NK1 receptor, HK-1 is also an agonist at the other tachykinin receptors[9], albeit with lower affinity. However, our previous results suggest that it may also have its own receptor [10–12]. HK-1, like other tachykinins, is expressed in the central nervous system (dorsal root ganglia, trigeminus, brainstem, cerebellum, cortex, and spinal cord), with the highest expression in the cerebellum and the periphery (thymus, heart, bone, skeletal muscle, stomach, lung, skin, and lactating breast) [13, 14], where it has various regulatory functions [15–17]. Its role has been demonstrated in the central nervous system, such as in pain [18, 19], stress responses [20] and depression-like behavior [20, 21].

Since HK-1 is present in high concentrations in the cerebellum[13], a major motor coordinator center, and detectable amounts in bone and skeletal muscle[22], the main aim of this study was to map the Tac4 gene localization throughout the motor coordination axis. Furthermore, we aimed to elucidate the function of HK-1 in the age-related deterioration of motor coordination and muscle strength. However, since HK-1 is expressed throughout the gonadal axis (hypothalamus, pituitary, and gonads) [13], exploring possible sex differences, with the help of male and female C57Bl/6 and Tac4-gene-deficient mice, was also a possible outcome of this study.

## Methods

### Detection of Tac4 mRNA in mouse and human samples with RNAscope technique

RNAscope in situ hybridization (ISH) was performed on 4-month-old male WT mice (*n* = 4). Animals were deeply anesthetized with an overdose of urethane (2.4 g/kg) and perfused transcardially with ice-cold 0.1 M phosphate-buffered saline (pH = 7.4) followed by 4% paraformaldehyde (PFA) in Millonig’s phosphate buffer. Dissected brains and spinal cords (SC) were postfixed for 72 h at 4 °C in PFA solution. The brains were coronally and the SC horizontally sectioned using a vibrating microtome (VT1000S, Leica Viosystems, Wetzlar, Germany). The 30 mm sections were stored in 1 × PBS with 0.01% Na-azide until further use (Merck KGaA, Darmstadt, Germany).

Perfused human cerebellar sample was obtained from one subject (Table 1) who died due to causes not directly involving any brain disease or damage, and without having medical history of neurological or psychiatric disorder either. All procedures were carried out in compliance with the Declaration of Helsinki and approved by the Regional Committee of Science and Research Ethics of Scientific Council of Health (ETT TUKEB 31443/2011/EKU, renewed: ETT TUKEB 15032/2019/EKU). Subject went through autopsy in the Department of Pathology of St. Borbála Hospital, Tatabánya. Informed consent by relatives was obtained for the use of brain tissue and for access to medical records for research purposes. The brain was removed after death, and the internal carotid and vertebrate arteries were cannulated. First, physiological saline containing 0.33% heparin was perfused through this system (1.5 L in 30 min), after which perfusion continued with Zamboni fixative solution containing 4% paraformaldehyde, and 0.2% picric acid in 0.1 M phosphate buffer (PB, pH = 7.4, 2 h, 4 L). Post-mortem interval (PMI) was determined between the time of death and the start of perfusion by Zamboni fixative. Frozen human somatomotor cortex, basal ganglia and spinal cord tissues were also obtained for RNAscope ISH (n = 4) in short post-mortem delay (1–12 h) without any major neuropathological alterations (Table 1). Tissue samples were microdissected in the Human Brain Tissue Bank, Semmelweis University. The human brain microdissection was approved by the Medical Research Council (ETT TUKEB); 5912–2/2018/EKU (Human Brain Tissue Bank, Semmelweis University, Budapest, Hungary), conducted with European directives and regulations. Informed consent was obtained from all participants and/or legal guardians. Following slow thawing of the frozen samples and 72 h of post-fixation, sectioning was performed [20] and processed as described earlier [23].

**Table 1:** Patient data (age, sex, time after death, and cause of death) of human brain samples

Subject No.	SKO29	#156	#166	#249	#255
Age	69 years	47 years	20 years	22 years	40 years
Sex	Male	Male	Male	Male	Male
Time after death	3.5 h	1 h	12 h	8 h	8 h
Cause of death	Acute myocardial infarction, unsp. (I21.9)	Cardiac insufficiency	Suicide	Suicide	Acute cardiorespiratory insufficiency

RNAscope ISH was performed using the RNAscope Multiplex Fluorescent Reagent Kit v2 (Advanced Cell Diagnostic (ACD), Newark, CA, USA) according to the manufacturer’s protocols. Mouse and human sections were hybridized with probes specific to mouse *Tac4* (ACD, Cat. No. 449651) or human *TAC4* (ACD, Cat. No. 896671), mouse *Vglut1* (ACD, Cat. No. 416631), human *VGLUT1* (ACD, Cat. No. 415611), mouse *Vglut2* (ACD, Cat. No. 319171), mouse *Gad1* (ACD, Cat. No. 400951), human *GAD1* (ACD, Cat. No. 404031), and mouse *Chat* (ACD, Cat. No. 408731) probes and were visualized by fluorescein (1:750 for *Chat*, *Vglut2, VGLUT1, GAD1*, and 1:3000 for *Vglut1*), cyanin3 (Cy3) (1:750 for *Tac4* and *TAC4*), and cyanin 5 (Cy5) (1:3000 for *Gad1*).

Mouse (ACD, Cat. No. 320881) and human (ACD, Cat. No. 320861) 3-plex positive control probes specific to *Polr2a* mRNA (fluorescein), *Ppib* mRNA (Cy3), and *Ubc* mRNA (Cy5) gave a well-recognizable signal in randomly selected sections of the areas of interest, while no signal was detectable with 3-plex negative (ACD, Cat. No. 320871) control probes upon hybridization and channel development (images not shown).

For expression studies on dopaminergic neurons in mice, some slides were further processed for immunofluorescence using rabbit anti-TH antibody (1:2000, Abcam, Cambridge, UK, Cat. No. Ab112). Superclonal Alexa Fluor 488 goat anti-rabbit (1:1000, Invitrogen Antibodies, Cat. No. A11034) was used as a secondary antibody.

All sections were counterstained with 4′,6-diamino-2-phenylindole (DAPI) and covered with ProLong Glass Antifade Mountant (Thermo Fisher Scientific, Waltham, MA, USA) for confocal imaging. Preparations were scanned by a Nikon Eclipse Ti2-E confocal microscope with 40x or 60x objectives. Virtual colors were selected to depict fluorescent signals: blue for DAPI; green for *Vglut1*, *Vglut2*, Chat, and TH; red for *Tac4/TAC4* and* GAD1*; and white for *Gad1*,* VGLUT1*, and* TAC4*. Brightness/contrast adjustment with maximum intensity of separate channels was processed using FIJI (version 1.53c, NIH, USA).

### RNA isolation and real-time quantitative polymerase chain reaction (RT-qPCR)

Total RNA isolation was performed using TRI-Reagent (Thermo Fischer Scientific, Waltham, MA, USA) and Direct-zol™ RNA MiniPrep (Zymo Research, Irvine, CA, USA) according to the manufacturer’s instructions. The concentration and purity of total RNA were determined using the Nanodrop ND-1000 Spectrophotometer V3.5 (NanoDrop Technologies, Inc., Wilmington, DE, USA). Samples were treated with 1 U DNase I enzyme to eliminate remaining genomic DNA. Tissue cDNA was synthesized with the Applied Biosystems™ High-Capacity cDNA Kit High-Capacity RT Kit (Thermo Scientific, Waltham, MA, USA). qPCR measurement of the mouse *Tac4* was carried out using the QuantStudio™ 5 system (Life Technologies Magyarország Ltd, Hungary), using the *eukaryotic translation elongation factor* (*Eef2*) and *glucuronidase beta* (*Gusb*) as reference genes. Measurements were performed in triplicates, in a reaction volume of 10 µl, containing 1x SensiFAST™ Probe Lo-ROX mix (Meridiane Bioscience, Memphis, USA), 400 nM probe primer mix (forward and reverse), and 20 ng cDNA. FAM-conjugated TaqMan™ Gene Expression Assays (Thermo Scientific, Waltham, MA, USA) were used to amplify the target loci, *Tac4*: Mm00474083_m1; *Eef2*: Mm00833287_g1; and *Gusb*: Mm01197698_m1. The geometric means of the Cq values were calculated for both reference genes, and the fold changes of *Tac4* were calculated using the ΔΔCt method [24, 25].

### Animals

We used young (3–4 months old), middle-aged (12 months old), and old (18 months old) male and female C57BL/6 and HK-1 deficient (*Tac4*^*–/–*^) mice weighing 25–50 g, bred and kept in the Laboratory Animal House of the Department of Pharmacology and Pharmacotherapy of the University of Pécs. Animals were housed at a constant temperature (24–25 °C), 50–60% relative humidity, a 12–12-h light–dark cycle, and free access to water and standard rodent food.

### Behavioral tests

All testing was conducted during the animals’ day cycle in an appropriately illuminated (400 lx) room. The animals were allowed to acclimate to the testing room for 30 min before the measurements. The mice were gently carried (e.g., in a strainer or in hand) from the cage to the testing apparatus. Four tests were used, which can be divided into two groups. The first group consisted of the rotarod and static bar tests, which examined dynamic and static motor coordination. The second group of tests, horizontal bar and grid tests, examined muscle strength. There was always a minimum of two days between each test, during which the animals were not exposed to any other influences. In all cases, only one test trial was performed and evaluated.

#### Rotarod test

Dynamic motor coordination was determined using a Rotarod apparatus (Ugo Basile, Italy). The rod rotates continuously and increasingly faster under the paws of the animals during the test, with a diameter of 3 cm and 30 cm above the base of the apparatus [10]. Two three-minute-long training sessions on the rod, which moves at a constant speed (10 rpm), were performed before the measurement. In the test trial, the starting speed was set to 10 rpm, the maximum rotation speed to 40 rpm, and the acceleration speed to 6 rpm/min. Mice could spend up to 300 s on the machine. The time spent on the rod was plotted and compared between the groups.

#### Static bar test

Static rod test measured static motor coordination. During the test, five wooden rods, each 60 cm long and of different thicknesses (35 (rod 1), 28, 22, 15, and 9 (rod 5) mm in diameter) were fixed to a laboratory shelf so that the rods extended horizontally into space. The height of the rods above the floor was 60 cm, and a soft cushion was placed underneath them to protect against a fall. We worked from the thickest rod to the thinnest. At the end of the pole, near the shelf, was the finish line, which was reached by turning from the side of the pole facing the space. The cut-off time was 120 s. Orientation time (the time required to turn around) and transit time (the time needed to reach the target line) were determined [26].

#### Horizontal bar test

The horizontal rod test assessed muscle strength. Two metal rods of different thicknesses were used, held horizontally by a wooden frame. They were 38 cm long and were held 49 cm above the surface of the bench by wooden support posts. The diameters of the poles were 4 and 6 mm. The test involved placing the animals at the midpoint of the pole by their front paws, from where they must climb onto the pole and touch the wooden frame with their nose or paws. They had 30 s to complete the task. Protective element(s) were placed under the pole, similar to the static pole test. The time required to complete the task/time on the bar was converted into scores. Shortly, a fall between 1–5 s meant 1 point, between 6–10 s = 2, 11–20 s = 3, 21–30 s = 4, and after 30 s = 5 points. Touching the bar with the forearm or nose without falling = 5 points [26]. The sum of the scores obtained on the two different bar thicknesses was the composite score.

#### Grid test

The grid test is a commonly used method for testing muscle strength [27]. In the experiment, animals were placed on a metal grid held by four poles 20 cm above the table. This grid is turned upside down, and the mice are then required to hold on to it using all four limbs for up to 180 s. The time spent on the grid was plotted and compared.

### Ethical consideration

Our testing methods and procedures comply with all requirements of Government Decree No. 40/2013 (14.II.) on the performance of animal experiments and the European Parliament's directives (2010/63). The University of Pécs Ethics Committee for Animal Experiments has approved the experimental protocols (Licence No. BA/73/00657-3/2022).

### Statistics

For the rotarod test, the static rod test, and the grid test, a two-way ANOVA with Fischer’s post hoc test was used. The results of the horizontal rod test were evaluated using the Kruskal–Wallis and Dunn’s post hoc test. Statistical analysis was performed using GraphPad Prism 8 software. Additionally, the effect sizes were also calculated using Hedges’ g (difference in means divided by the pooled and weighted standard deviation) in all relations. Given that the study was conducted on a small sample size, the statistical significance level (*p*-value) alone could not provide reliable information. Hence, we used effect size to interpret the results, providing a more direct picture of the magnitude and practical significance of the effect [28]. An effect size > 0.2 was considered small, >0.5 as medium, and >0.8 as large [29]. The results for each group were presented as mean ± SEM. The number of elements per group was 8–25.

## Results

### Tac4 expression in mouse and human motor coordinating system

**Fig. 1:**
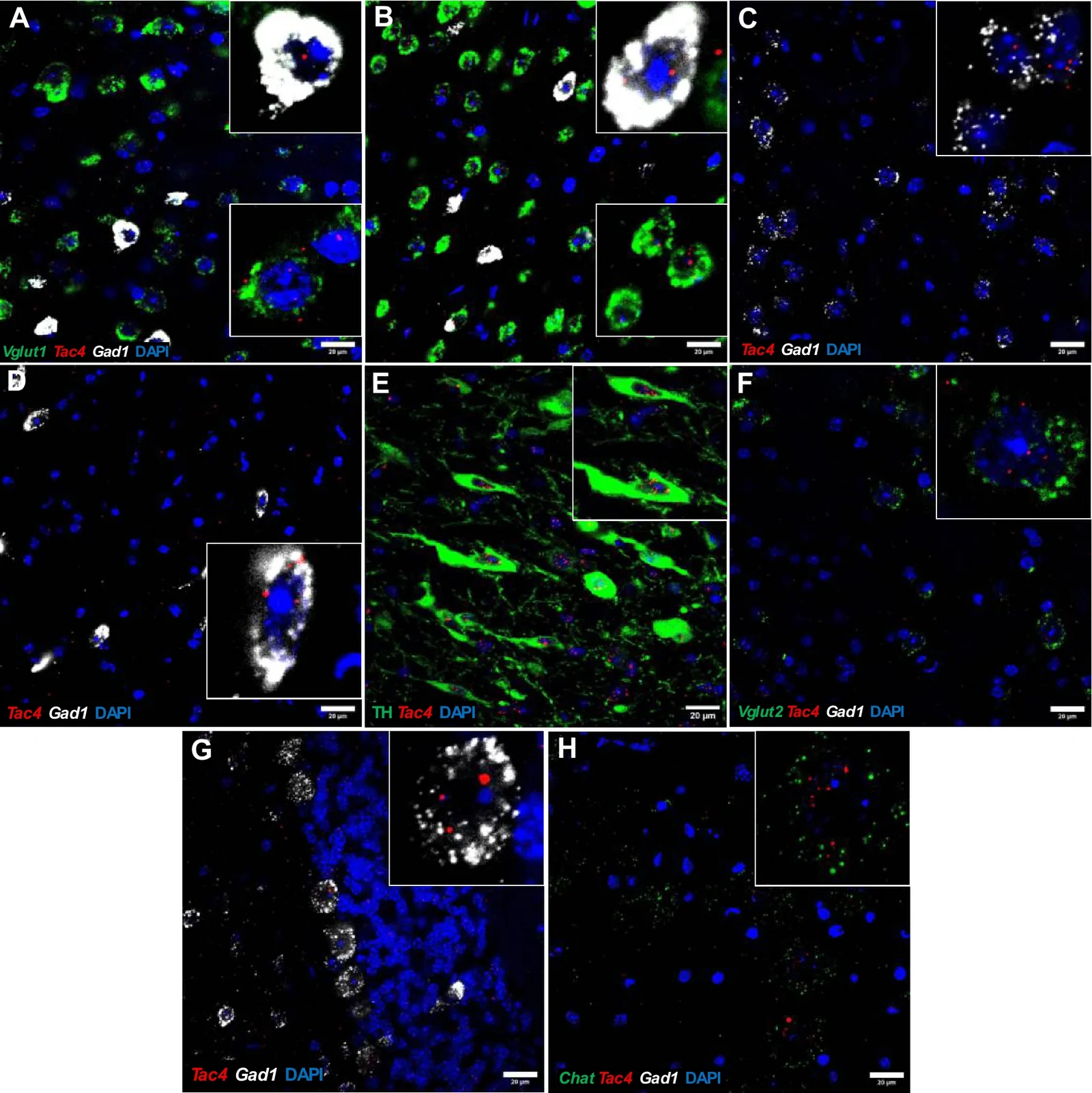
Representative images of *Tac4* expression in murine brain regions involved in motor coordination. Colocalization of *Tac4* mRNA (red) with glutamatergic (*Vglut1* or *Vglut2*, green), GABAergic (*Gad1*, white), dopaminergic (TH, green), and cholinergic (*Chat*, green) neurons is observed in the M1 (**A**) and M2 (**B**) motor cortex, caudoputamen (**C**), medial globus pallidus (**D**), substantia nigra pars reticulata (**E**), ventral thalamic nucleus (**F**), cerebellum (**G**), and ventral horn of the spinal cord (**H**). Scale bars: 20 mm

Mouse and human *Tac4* mRNA expression was investigated using RNAscope ISH in various brain regions and the spinal cord, which are involved in motor coordination. Confocal imaging revealed that *Tac4* expression is detectable in *Vglut1*-expressing excitatory and *Gad1*-expressing inhibitory neurons in the M1 (Fig. 1A) and M2 (Fig. 1B) regions of the motor cortex. Tac4 expression was also observed within distinct nuclei of the basal ganglia, Gad1^+^ inhibitory interneurons of the caudoputamen (Fig. 1C) and medial globus pallidus (Fig. 1D), as well as dopaminergic neurons of the substantia nigra pars reticulata (Fig. 1E), which clearly expressed the gene. Co-expression of *Tac4* and *Vglut2* was observed in neurons of the ventral thalamic nucleus (Fig. 1F). Cerebellar inhibitory Purkinje cells, essential for motor coordination, also express *Tac4* (Fig. 1G). The presence of *Tac4* mRNA was also confirmed in cholinergic motor neurons in the ventral horn of the lumbar spinal cord (Fig. 1H).

**Fig. 2:**
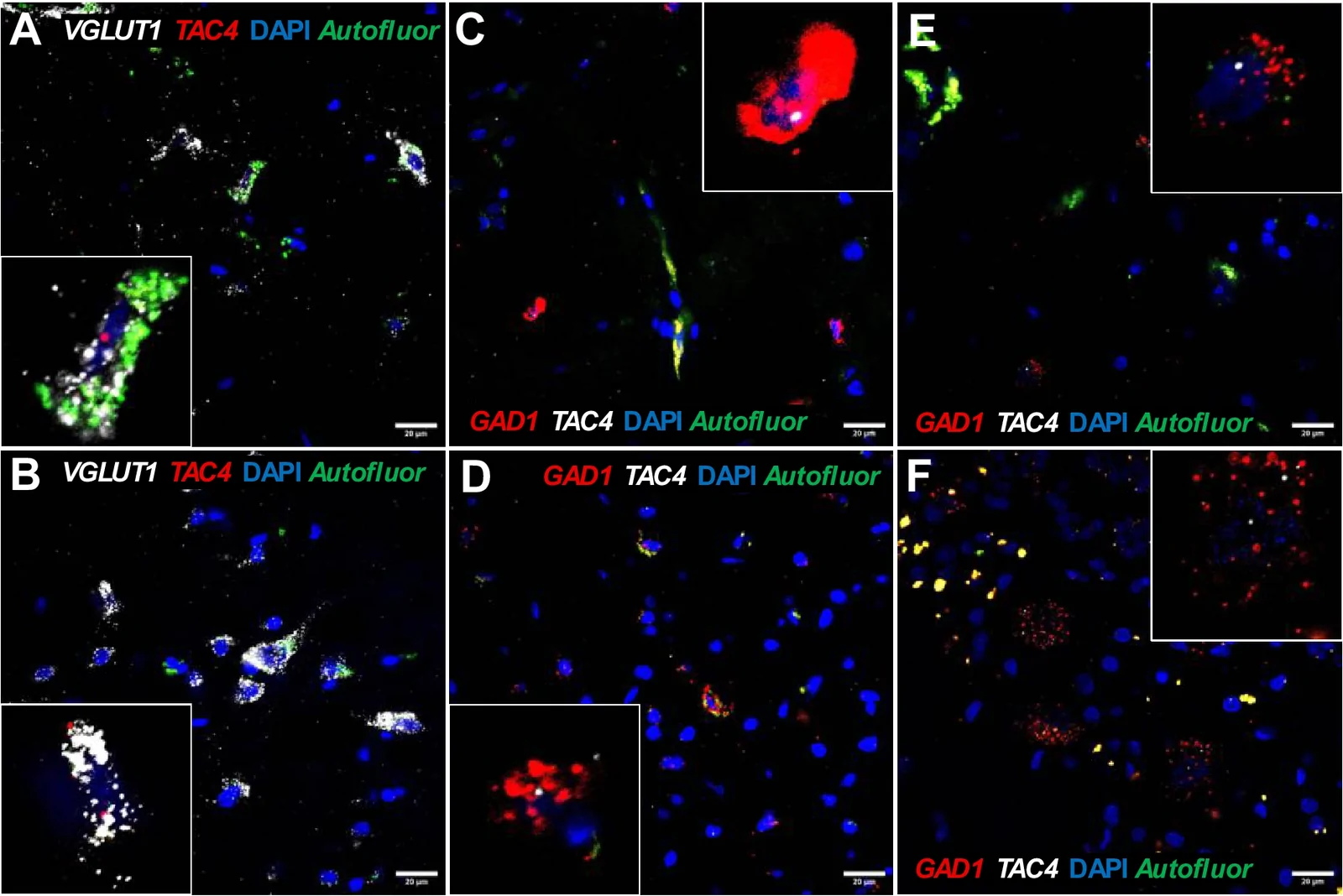
Representative images of *TAC4* expression in human brain samples. In the motor cortex, colocalization of *TAC4* (red or white) can be observed in glutamatergic (*VGLUT1*, white) (**A, B**) and GABAergic (*GAD1*, red) (**C, D**) neurons from samples of different patients. Inhibitory neurons (red) in the putamen (**E**) and Purkinje cells (red) in the cerebellum (**F**) also express *TAC4* mRNA (white)

Data from human tissue experiments further corroborate the expression of the *TAC4* gene within the motor cortex, both in VGLUT1^+^—excitatory (Fig. 2A, B) and GAD1^+^-inhibitory neurons (Fig. 2C, D). The presence of *TAC4* mRNA was observed in inhibitory neurons in the putamen of the basal ganglia (Fig. 2E), and in cerebellar Purkinje cells (Fig. 2F). The green signal detected in the human tissues was determined to be a result of autofluorescence.

### Tac4 expression increases with age in the cerebellum and decreases in the muscle

**Fig. 3:**
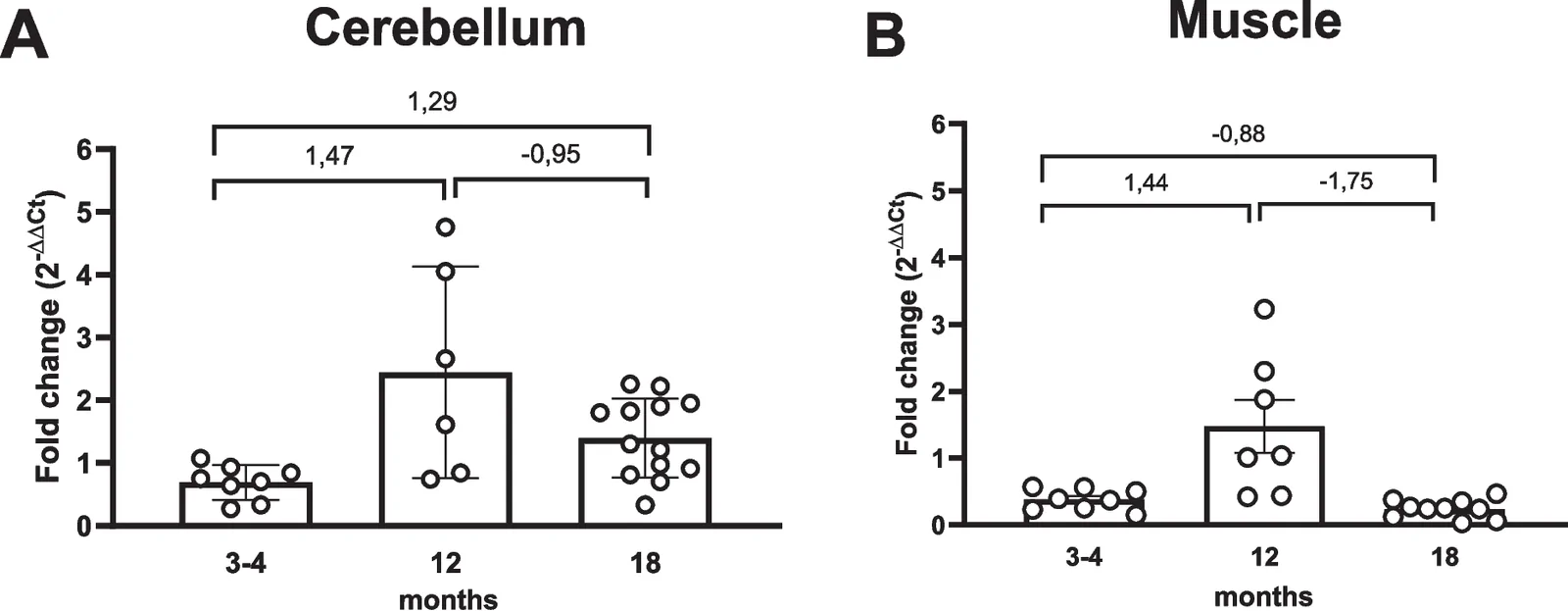
Fold change of Tac4 gene expression compared to *Eef2* and *Gusb* reference genes in **A** cerebellum and **B** muscle samples of 3 to 4-, 12-, and 18-month-old wildtype mice (*n* = 6–13). Statistical analysis was performed using effect size analysis, medium effect size: Hedge’s *g* ≥ 0.5; large effect size: Hedge’s g ≥ 0.8, indicating differences between age groups

In agreement with the RNAscope ISH results, *Tac4* mRNA is stably expressed in mouse cerebellum and striated muscle in all investigated age groups. In 18-month-old mice, an approximately 1.5-fold increase was measured, while in 12-month-old animals, an even higher, 3.5-fold elevation of Tac4 mRNA was detectable in cerebellar samples (Fig. 3A). Tac4 mRNA level of the 12-month-old group was also the highest in muscle samples (Fig. 3B).

### Motor coordination deteriorates with age and sex

**Fig. 4:**
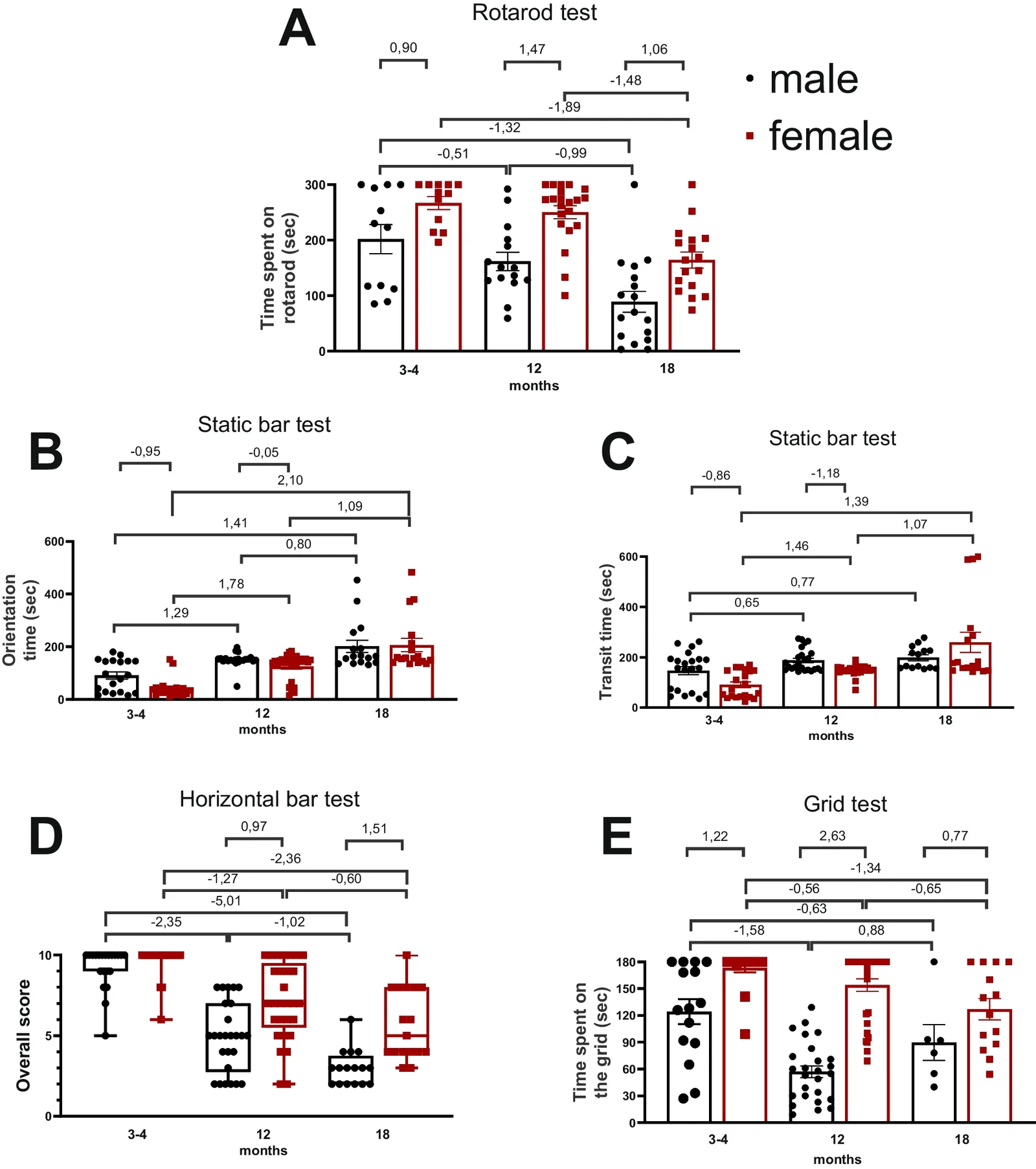
Age-related motor coordination changes measured in rotarod test (**A**), static bar test–orientational time (**B**), static bar test–transit time (**C**), and muscle strength loss assessed in horizontal bar test (**D**) and grid test (**E**) of wildtype male and female, 3 to 4-, 12-, and 18-month-old mice. Data points demonstrate the means ± SEM of *n* = 10–25 per group. Statistical analysis was performed using effect size analysis, medium effect size: Hedge’s *g* ≥ 0.5; large effect size: Hedge’s *g* ≥ 0.8 indicating differences between age groups or between female and male groups. Two-way ANOVA followed by Fisher’s post hoc test was also performed. The *F* values for the tests were the following: rotarod test (**A**)—*F* (2, 88) = 0.2421, static bar test–orientational time (**B**)—*F* (2, 117) = 1.556, static bar test—transit time (**C**)—*F* (2, 119) = 5.963, horizontal bar test (**D**)—*F* (2, 109) = 3.381, and grid test (**E**)—*F* (2, 103) = 5.940. The figure shows Hedge’s *g* scores

Aging significantly reduced motor coordination in both sexes. In the Rotarod test, females performed significantly better than males in all three age groups. Older animals performed significantly worse than younger animals. They spent less time on the machine, which suggests weaker motor coordination skills (Fig. 4A). Similarly, older animals had higher orientation and transit times in the static bar test, meaning that motor coordination is deteriorated (Fig. 4B, C) (The p values are shown in Supplementary Table 1; Hedge’s values are shown in Supplementary Table 2).

### Muscle strength weakens with age

Our studies have shown that muscle strength decreases with age in both males and females. In the horizontal bar test, older animals had significantly higher overall scores (Fig. 4D), while in the grid test, they spent significantly less time on the grid (Fig. 4E) compared to the young ones. Interestingly, the 18-month-old males performed better than the 12-month-old group. Muscle strength tests also revealed sex differences, with females performing significantly better than males, demonstrating less muscle deterioration in females compared to males. (The *p* values are shown in Supplementary Table 1; Hedge’s values in Supplementary Table 2.)

### Hemokinin-1 deficiency affects motor coordination

**Fig. 5:**
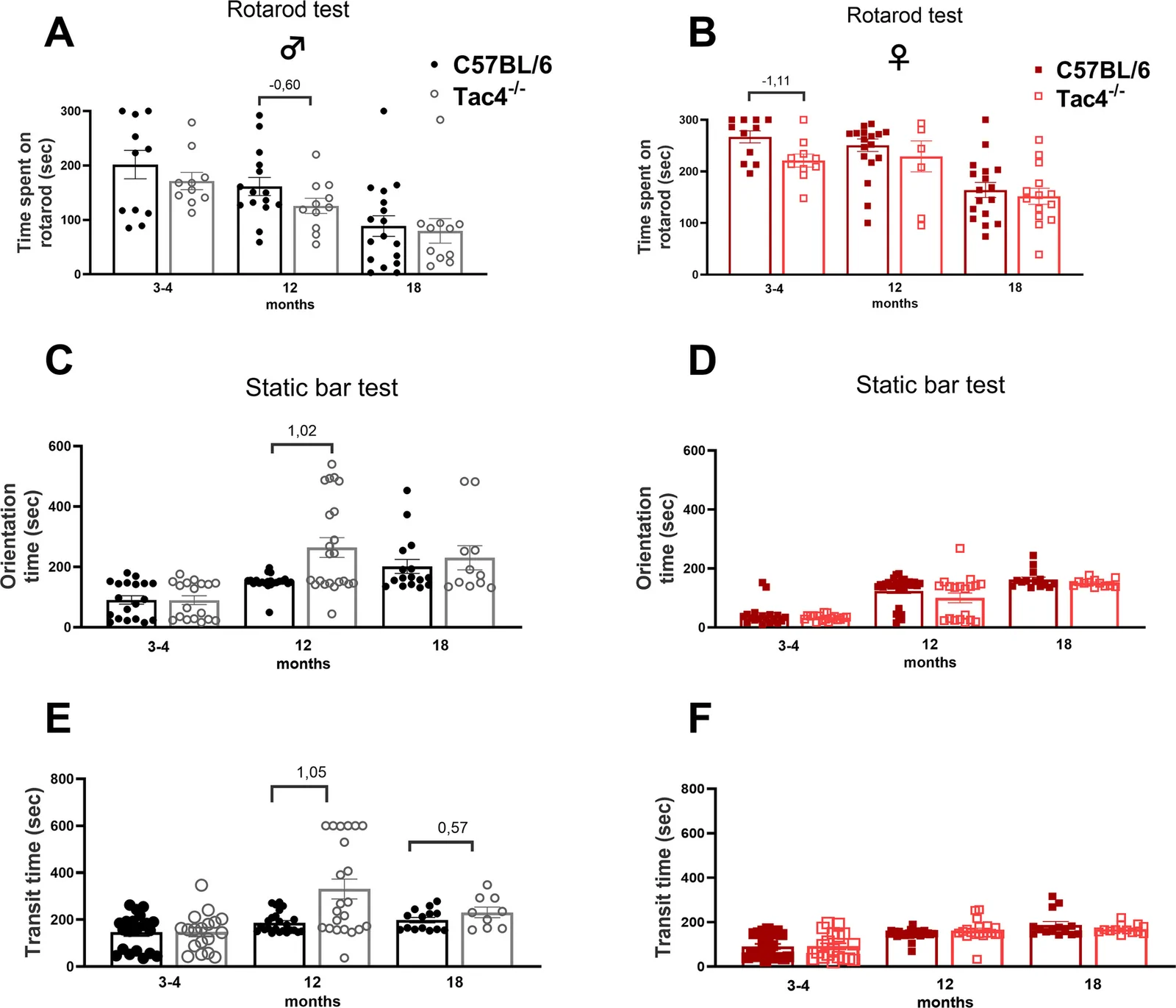
Changes of motor coordination in rotarod (**A, B**) and static bar (**C–F**) tests of HK-1-deficient (Tac4^−/−^) mice compared to WT. Data showed of male and female, 3 to 4-, 12-, and 18-month-old mice. Data points demonstrate the means ± SEM of n = 10–25 per group. Statistical analysis was performed using effect size analysis. *Medium effect size: Hedge’s *g* ≥ 0.5; **large effect size: Hedge’s *g* ≥ 0.8 indicating differences between C57Bl/6 and Tac4^−/−^ groups. Two-way ANOVA followed by Fisher’s post hoc test was also performed. The *F* values for the tests were the following: rotarod test for male (**A**)—*F* (2, 70) = 0.2633, rotarod test for female (**B**)—*F* (2, 76) = 0.5796, static bar test–orientational time for male (**C**)—*F* (2, 102) = 3.886, static bar test–orientational time for female (**D**)—*F* (2, 110) = 0.4403, static bar test–transit time for male (**E**)—*F* (2, 102) = 5.176, and static bar test–transit time for female (**F**)—*F* (2, 112) = 1.94. The figure shows Hedge’s *g* scores

We detected a significant difference in the rotarod test (Fig. 5A, B), as our results show that middle-aged male mice performed significantly worse, spending less time on the machine, indicating poorer motor coordination. The outcome was similar in the static rod test. In aged males (Fig. 5C, E), we observed higher orientation and transit times in the case of HK-1 deficiency, indicating impaired motor coordination. In young females (Fig. 5D, F), gene deficiency also decreased motor coordination skills measured in the rotarod test. (The *p* values are shown in Supplementary Table 3; Hedge’s values are shown in Supplementary Table 4.)

### Hemokinin-1 age- and sex-dependently influences muscle strength

**Fig. 6:**
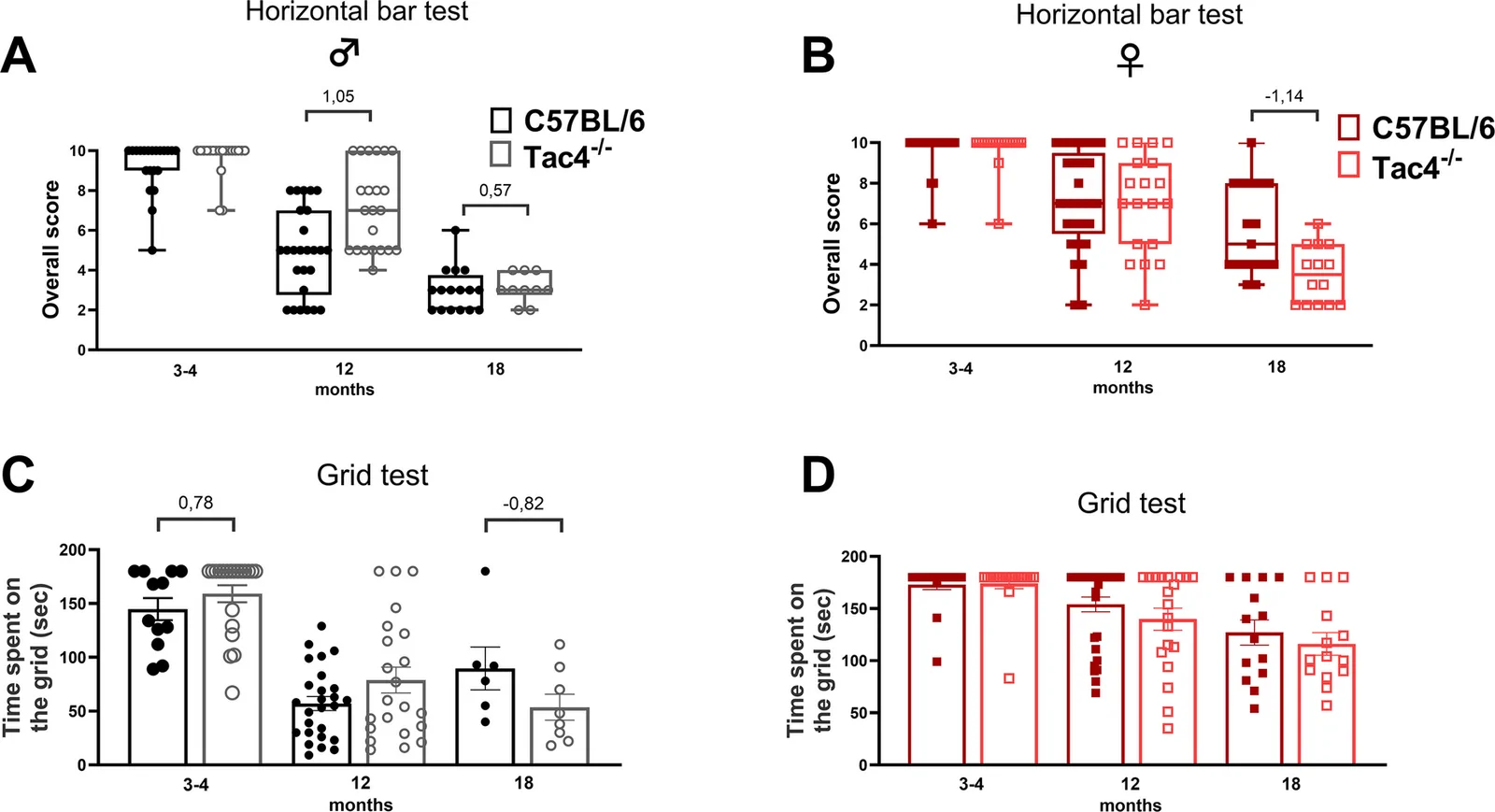
Changes of muscle strength in horizontal bar (**A, B**) and grid (**C, D**) tests of HK-1-deficient (Tac4^−/−^) mice compared to WT. Data showed of male and female, 3 to 4-, 12-, and 18-month-old mice. Data points demonstrate the means ± SEM of *n* = 15–25 per group. Statistical analysis was performed using effect size analysis. *Medium effect size: Hedge’s *g* ≥ 0.5; **large effect size: Hedge’s *g* ≥ 0.8 indicating differences between C57Bl/6 and Tac4^−/−^ groups. Two-way ANOVA followed by Fisher’s post hoc test was also performed. The *F* values for the tests were the following: Horizontal Bar test for male (**A**)—*F* (2, 109) = 3.381, horizontal bar test for female (**B**)—*F* (2, 117) = 1.556, grid test for male (**C**)—*F* (2, 91) = 3.3230, and grid test for female (**D**)—*F* (2, 109) = 0.4613. The figure shows Hedge’s g scores

HK-1-deficient 3- and 12-month-old male mice performed significantly better in the grid test and horizontal bar test (Fig. 6A, C), indicating greater muscle strength than WT animals. Interestingly, 18-month-old WT males and females showed higher scores and more time spent on the grid compared to the knockouts (Fig. 6B, C). (The *p* values are shown in Supplementary Table 3; Hedge’s values are shown in Supplementary Table 4.)

## Discussion

These are the first data describing the widespread expression of HK-1 in glutamatergic, GABAergic, dopaminergic, and cholinergic neuronal populations in motor coordination centers, such as the primary and secondary motor cortices, basal ganglia, and cerebellum, in both mouse and human brains, suggesting the translational relevance of the mouse functional results. HK-1 exerts age- and sex-dependent effects on motor coordination with a predominantly protective action on aging-induced decline in males. Meanwhile, its function in muscle strength regulation is more complex, with a protective role only in old age, potentially due to its distinct effects centrally and peripherally.

Additionally, we present a detailed comparison of age-associated motor functions in a sex-dependent manner.

HK-1 is present in the human and mouse primary motor cortex, which acts as a central controller of motor functions [30, 31]. Furthermore, it is also abundantly expressed in the dopaminergic cells of the substantia nigra (SN), part of the nigrostriatal system. SN is involved in both motor coordination and cognitive processes, and it has strong connections with other brain areas, such as the. Previous data demonstrated a high HK-1 expression [13, 20] in the cerebellum, an important regulator of cognitive, social, emotional, and motor functions [34, 35]. We also proved HK-1 expression in this brain region, especially in Purkinje cells.

There are only a few papers in the literature demonstrating age- and sex-dependent motor coordination functions in mice[36–38]. Based on Deacon et al., 2013, we set up a complex functional assessment panel using the Rotarod, static and horizontal bars, as well as the grid test, which proved to be appropriate to demonstrate age-related motor coordination and muscle strength changes in both male and female mice. C57Bl/6 mice showed progressively worse coordination and strength by the age of 18 months in both sexes. However, females performed significantly better in all tests in almost all age groups. Literature data are inconsistent regarding sex-dependent motor coordination. This finding aligns with recent data indicating better performance in female mice compared to age-matched males [36]. Some publications indicate no differences between young male and female mice in the rotarod test [39], while others demonstrate minor sex- and age-dependent differences [40, 41]. Females learn faster, potentially due to estrogen dominance during the reproductive period [42]. The mouse estrous cycle begins about 26 days after birth [43] and continues for the rest of their lives [44], but usually becomes irregular after the age of 17 months [45]. These age- and sex-dependent motor function changes are similar to the minor differences described in humans in the literature [46]. Age was found to be a more critical determinant of motor function decline than gender [47, 48].

We detected better muscle function in young female mice in the grid test, which showed later and smaller deterioration than males. However, greater muscle strength in the grip test was demonstrated in male mice [39], but this test measures the maximum force displayed by an animal, not like in the grid test, where the time spent on the grid is determined. A limitation of the grid test is that in many cases, it is difficult to judge whether an animal falls off the grid because it cannot hold itself or, especially in younger animals, because it wants to escape an uncomfortable position.

Both horizontal bar and grid tests showed age-dependent muscle strength and inter-muscular coordination impairment, similar to humans [2] above the age of 30 [49]. Male muscle strength is generally greater than that of females [50], but it similarly declines with age [51]. However, recent data also suggest an accelerated reduction of peripheral neuromuscular function in females in the fourth decade around menopause, which can be attributed to altered body composition, physical activity, and sex hormones [52].

These results indicate that HK-1 is involved in maintaining normal motor coordination and exhibits a complex regulatory function in muscle strength. One-year-old male HK-1-deficient animals showed significantly higher orientation and transit times, as well as time spent on RotaRod, indicating impaired locomotor coordination. However, in females, such a difference only appeared at 3 months of age. The fundamental role in locomotor coordination is likely to be attributed to the central effect of HK-1, which is supported by its high expression in the cerebellum, increasing with aging.

Interestingly, in 12-month-old males, HK-1 deficiency resulted in better muscle strength despite worse motor coordination. These two parameters cannot be independently investigated, although coordination only partially depends on muscle function; complex central nervous system mechanisms greatly contribute to this parameter [53–55]. HK-1 expression has been described in skeletal muscles in humans and in mice [56]; (https://www.proteinatlas.org/ENSG00000176358-TAC4/tissue#rna_expression), which seems to have deteriorating local function in contrast to the brain, presumably mediated by distinct receptors. Since HK-1 expression declines with aging in soleus muscle, it might have a peripheral direct action on muscle strength. This could potentially target the mitochondria [57] since HK-1 was demonstrated to influence a range of mitochondrial gene expressions [58].

Furthermore, another hypothesis for the sex differences might be the modulatory effect of estradiol on cerebellar glutamatergic neurotransmission [59, 60] and/or a potential interaction between HK-1 and the kisspeptin-neurokinin B (NKB) system involved in reproductive processes [61]. Both NKB and HK-1 tachykinins can bind to the NK2 receptor [62] playing a role in the secretion of Kisspeptin on KNDy neurons [63, 64]. Testosterone and estradiol levels decline with aging in both humans [65] and rodents [66] leading to various central nervous system malfunctions [67]. All this suggests that HK-1, potentially through interactions with NKB and NK2 receptors, may influence gonadal axis hormones, ultimately plasma levels of sex hormones, resulting in sex differences.

In conclusion, we demonstrated that the HK-1 is widely expressed in glutamatergic, GABAergic, dopaminergic, and cholinergic neurons throughout the entire motor coordinating system. HK-1 plays a predominantly protective role in age-related motor coordination decline, particularly in males. Identifying its targets and signaling pathways could open novel perspectives for improving age-related motor functions.

## Supplementary Information

Below is the link to the electronic supplementary material.

ESM 1 (DOCX 27.1 KB)

## Data Availability

The authors confirm that the data supporting the findings of this study are available within the article and its supplementary materials.
