# Supplementary material for: Widespread neuronal hemokinin-1 expression in motor regions and its protective role in age-related impairment
Source: GeroScience. 2025 Nov 12;48(4):5941–55. doi: 10.1007/s11357-025-01972-4 (PMC13575092; doi:10.1007/s11357-025-01972-4)
Supplement: Supplementary file 1 [file 11357_2025_1972_MOESM1_ESM.docx]

|  | 3-4 vs 12 months old (male) | 3-4 vs 18 months old (male) | 12 vs 18 months old (male) | 3-4 vs 12 months old (female) | 3-4 vs 18 months old (female) | 12 vs  18 months old (female) | 3-4 months old  male vs female | 12 months old  male vs female | 18 months old  male vs female |
| --- | --- | --- | --- | --- | --- | --- | --- | --- | --- |
| Time spent on Rotatod | 0.1435 | **<0.0001** | **0.0046** | 0.4234 | **<0.0001** | **<0.0001** | **0.0171** | **0.0001** | **0.0012** |
| Orientation time (Static rods) | 0.0517 | **0.0010** | 0.1052 | **<0.0001** | **<0.0001** | **0.0001** | **0.0206** | 0.1602 | 0.8245 |
| Trasition time (Static rods) | 0.2090 | 0.1669 | 0.7609 | **0.0237** | **<0.0001** | **<0.0001** | **0.0188** | 0.0599 | **0.0285** |
| Composite score (Horizontal bars) | **<0.0001** | **<0.0001** | 0.5622 | **0.0056** | **<0.0001** | 0.9177 | >0.9999 | **0.0429** | 0.1273 |
| Time spent on the grid | **<0.0001** | 0.1099 | 0.1072 | 0.0875 | **0.0007** | **0.0264** | **0.0005** | **<0.0001** | 0.0527 |

**Supplementary Table 1.** Statistical results (*p* values) of WT male and female animals based on two-way ANOVA followed by Fischer’s posttest in behavior measurements and Kruskal-Wallis test followed by Dunn’s posttest in horizontal rod test. Significant alterations are indicated in bold.

|  | 3-4 vs 12 months old (male) | 3-4 vs 18 months old (male) | 12 vs 18 months old (male) | 3-4 vs 12 months old (female) | 3-4 vs 18 months old (female) | 12 vs  18 months old (female) | 3-4 months old  male vs female | 12 months old  male vs female | 18 months old  male vs female |
| --- | --- | --- | --- | --- | --- | --- | --- | --- | --- |
| Time spent on Rotatod | **-0.51** | **-1.32** | **-0.99** | -0.32 | **-1.89** | **-1.48** | **0.90** | **1.47** | **1.06** |
| Orientation time (Static rods) | **1.29** | **1.41** | **0.80** | **1.78** | **2.10** | **1.09** | **-0.95** | **-0.57** | **0.05** |
| Trasition time (Static rods) | **0.65** | **0.77** | 0.24 | **1.46** | **1.39** | **1.07** | **-0.86** | **-1.18** | 0.47 |
| Composite score (Horizontal bars) | **-2.35** | **-5.01** | **-1.02** | **-1.27** | **-2.36** | **-0.60** | 0.34 | **0.97** | **1.51** |
| Time spent on the grid | **-1.58** | **-0.63** | **0.88** | **-0.56** | **-1.34** | **-0.65** | **1.22** | **2.63** | **0.77** |

**Supplementary Table 2.** Statistical results (*hedges g values*) of WT male and female animals based on effect sizes. Significant alterations are indicated in bold.

|  | **Male** | | | **Female** | | | |
| --- | --- | --- | --- | --- | --- | --- | --- |
|  | 3-4-month-old C57Bl/6 vs Tac4 KO | 12-month- old C57Bl/6 vs Tac4 KO | 18-month- old C57Bl/6 vs Tac4 KO | | 3-4-month-old C57Bl/6 vs Tac4 KO | 12-month- old 57Bl/6 vs Tac4 KO | 18-month- old C57Bl/6 vs Tac4 KO |
| Time spent on Rotatod | 0.3176 | 0.2041 | 0.7428 | | 0.0613 | 0.3571 | 0.5495 |
| Orientation time  (Static rods) | 0.9784 | **0.0001** | 0.4480 | | 0.6985 | 0.2216 | 0.1047 |
| Trasition time (Static rods) | 0.9866 | **<0.0001** | 0.4662 | | 0.9801 | 0.5327 | 0.0519 |
| Composite score (Horizontal bars) | >0.9999 | **0.0403** | >0.9999 | | >0.9999 | >0.9999 | 0.3997 |
| Time spent on the grid | **0.0229** | 0.0916 | 0.1352 | | 0.9318 | 0.1884 | 0.4377 |

**Supplementary Table 3.** Statistical results (*p* values) of WT and Tac4^-/-^ (KO) animals based on two-way ANOVA followed by Fischer’s posttest in behavior measurements and Kruskal-Wallis test followed by Dunn’s posttest in horizontal rod test. Significant alterations are indicated in bold.

|  | **Male** | | | **Female** | | |
| --- | --- | --- | --- | --- | --- | --- |
|  | 3-4-month-old C57Bl/6 vs Tac4 KO | 12-month- old C57Bl/6 vs Tac4 KO | 18-month- old C57Bl/6 vs Tac4 KO | 3-4-month-old C57Bl/6 vs Tac4 KO | 12-month- old 57Bl/6 vs Tac4 KO | 18-month- old C57Bl/6 vs Tac4 KO |
| Time spent on Rotatod | -0.39 | **-0.60** | -0.11 | **-1.11** | -0.33 | -0.20 |
| Orientation time  (Static rods) | -0.01 | **1.02** | 0.25 | -0.30 | -0.40 | -0.38 |
| Trasition time (Static rods) | 0.01 | **1.05** | **0.59** | 0.01 | 0.45 | -0.40 |
| Composite score (Horizontal bars) | 0.33 | **1.05** | **0.57** | 0.10 | -0.04 | -**1.14** |
| Time spent on the grid | **0.78** | 0.48 | **-0.82** | 0.05 | -0.33 | -0.25 |

**Supplementary Table 4.** Statistical results (*hedges g values*) of WT and Tac4^-/-^ (KO) animals based on effect sizes. Significant alterations are indicated in bold.
